# Transrectal Prostate Biopsy Approach in Men Undergoing Kidney Transplant: A Retrospective Cohort Study at Three Referral Academic Centers

**DOI:** 10.3390/diagnostics14030266

**Published:** 2024-01-25

**Authors:** Lucio Dell’Atti, Viktoria Slyusar, Piero Ronchi, Stefano Manno, Chiara Cambise

**Affiliations:** 1 Department of Urology, University-Hospital of Marche, 60126 Ancona, Italy; piero.ronchi@ospedaliriuniti.marche.it; 2 Pain Therapy Center, Division of Anesthesia and Intensive Care, University-Hospital of Marche, 60126 Ancona, Italy; viktoria.slyusar@ospedaliriuniti.marche.it; 3 Department of Urology, University-Hospital Renato Dulbecco, 88100 Catanzaro, Italy; stefanomanno86@gmail.com; 4 Department of Emergency, University-Hospital Gemelli IRCSS, 00168 Roma, Italy; chiara.cambise@policlinicogemelli.it

**Keywords:** prostate cancer, prostate biopsy, transrectal, transperineal, MRI-guided, complications, kidney transplant, outcomes

## Abstract

Background: Currently, there are no studies evaluating the feasibility of a prostate biopsy approach in men undergoing a kidney transplant (KT). Owing to this evidence, we planned a retrospective population-based study to evaluate our experience of a transrectal prostate biopsy (TR-PB) approach and studied the impact on the complication rate and outcomes in patients undergoing KT with suspected prostate cancer (PCa). Methods: We collected data from KT patients who underwent PB with a transrectal approach. One week and two weeks after the PB, patients’ information was collected regarding possible complications during the post-biopsy period. Results: A total of 121 patients were included in this study. Among them, Group 1 was composed of 59 patients undergoing TR-PB with an ultrasound (US) standard technique, and Group 2 consisted of 62 patients undergoing TR-PB with an MRI-US cognitive technique. We observed a 28.9% Clavien–Dindo grade ≤ 2 of early side effect rates (mostly rectal bleeding and other minor hematuria), with a very low rate of hospital re-admission for acute urinary retention (3.3%); only one man required hospitalization for rectal bleeding, and there were no major complications. Conclusions: We can affirm that TR-PB can be a safe procedure with a low risk of severe complications when performed by skilled specialists with a standardized procedural pathway.

## 1. Introduction

The kidney transplant (KT) has grown exponentially in recent years, as the extension of organ transplantation eligibility criteria has allowed older patients to benefit from KT [1]. Prostate cancer (PCa) is among the most common tumors in men. The average age of patients diagnosed with PCa is approximately 65 years. About 33% of patients between the ages of 60 to 70 years will harbor PCa, and approximately 3% will die of this cancer [2]. Therefore, the larger parts of PCa are indolent and are usually undiscovered and untreated. The latest KDIGO clinical guidelines for KT candidates state that Grade Group 1 of PCa is one condition where KT candidates with an active malignancy do not need to be omitted from transplantation programs [3]. A prostate biopsy (PB) is the gold standard approach to diagnose PCa. It is performed with a transrectal or transperineal technique [4]. Recently, magnetic resonance imaging (MRI), with a cognitive or ultrasound (US) fusion technique, has started to be used for targeted biopsies to increase the detection rate of clinically significant prostate cancer (csPCa) [5]. Currently, there are no studies evaluating the feasibility of the transrectal prostate biopsy (TR-PB) approach in KT men. Owing to this evidence, we planned a retrospective population-based study to evaluate our clinical experience in a TR-PB approach and study the impact on the complication rate and outcomes in patients undergoing KT with suspected PCa.

## 2. Materials and Methods

### 2.1. Study Design and Selection Criteria

We retrospectively collected data from KT patients (≥12 months) who underwent PB with a transrectal approach at three referral academic centers from January 2010 to November 2023. The inclusion criteria were: men (>18 years) undergoing KT for end-stage renal disease and scheduled for suspected PCa (increased PSA levels and/or suspected digital rectal examination [DRE] and/or multiparametric MRI [mpMRI] with suspicious lesions [PIRADS ≥ 3]) and patients eligible for TR-PB with a US standard technique or MRI-US cognitive technique. The exclusion criteria were: <18 years, men undergoing KT < 12 months (for reducing the risk of post-procedural infection), instable graft function, patients with no access to the rectum (e.g., history of anorectal surgery and/or colorectal diseases), patients whose procedure required general anesthesia, patients who previously had prostate surgical treatments and patients with a positive urine culture (the urine culture was considered positive if there were >105 colony forming units/mL of ≥1 bacterial strain). All TR-PB were performed by three expert urologists, using a transrectal end-fire transducer equipped with a disposable needle guide kit. One week and two weeks after the PB, patients’ information was collected regarding possible complications during the post-biopsy period. All adverse events that occurred as a consequence of the procedures were described in the patient’s medical records. The complications of interest were: pain requiring analgesics post-biopsy, hematuria, rectal bleeding, urinary tract infections (UTIs), sepsis and/or acute urinary retention (AUR). All biopsy samples were interpreted by dedicated uro-oncological pathologists. All enrolled patients signed the informed consent form.

### 2.2. Treatment Protocols

We used an ultrasound machine equipped with a 5–9 MHz multi-frequency convex probe “end-fire” for standard and cognitive TR-PBs. All procedures were executed in order to empty the bladder, and the patient was in the left lateral decubitus. Prostate biopsies were performed transrectally in an outpatient setting under local anesthesia with a prostate perineural block under US guidance (10 mL of 1% lidocaine and injection of 5 mL on each side of the prostate gland). Patients on anticoagulation/antiplatelet therapy were considered eligible for the study, providing they had followed the instructions of stopping antiplatelet drugs at least 5 days before the PB and changed them with low molecular weight heparin before the procedure. Antibiotic prophylaxis was administered before the procedure and consisted of received one prophylactic dose of Ceftriaxone (1 g) 2 h before the PB. In patients where an allergy to Cefalosporine was reported, prophylaxis with Levofloxacin, 500 mg orally, was administered. A cleansing rectal enema was self-performed at home by each patient in the morning of the procedure and a povidone-iodine rectal enema was self-administered 10 min before the TR-PB. The TR-PB standard protocol was a combined scheme that includes 12 cores for prostate volume (PV) ≤ 30 mL, 14 cores for PV > 30 mL but <60 mL and 18 cores for PV ≥ 60 mL [6]. A cognitive biopsy was performed using MRI to identify a zone of doubt within the prostate and subsequent targeting of the lesions under US guidance without the use of a software machine for the recording of the MRI and US images. The urologist reviewed the MRI to define the position of the suspicious lesion and then used anatomic landmarks to correlate the lesion location to a site on the US images of the biopsy. The zone of the lesion was considered in relation to landmarks such as prostatic zones, the urethra or cysts. However, if a correlation on US was identified, this correlation was directly targeted. The protocol for an mpMRI-US cognitive biopsy was a combined scheme that included a targeted biopsy (4 cores) in addition to a standard TR-PB scheme (6 cores for PV ≤ 30 mL, 8 cores for PV > 30 mL but <60 mL, and 10 cores for PV ≥ 60 mL) to improve the detection of clinical csPCa.

### 2.3. Statistical Analysis

Continuous variables were illustrated as medians and interquartile ranges and nominal variables as numbers and percentages. Comparisons between two groups were performed using the Chi-square test or Fisher’s exact test for discrete variables and the Mann–Whitney U test for continuous variables. A value of *p* < 0.05 was considered statistically significant. All statistical analyses were conducted using SPSS version 24 (IBM Corp., Armonk, NY, USA)

## 3. Results

A total of 127 KT patients suspicious for PCa underwent a TR-PB protocol and were enrolled in the study. Among them, six patients (4.9%) were lost to follow-up and thus were excluded.

A total of 121 medical records of patients were included in the study. All men undergoing KT had a stable graft function, absence of immunodeficiency, or complex metabolic conditions (such as the presence of uremia, malnutrition, poorly controlled diabetes mellitus and cirrhosis). Among them, Group 1 (G1) was composed of 59 patients that received TR-PB with a US standard technique, and Group 2 (G2) consisted of 62 patients that received TR-PB with an MRI-US cognitive technique. Patients’ characteristics at inclusion are indicated in Table 1 and Table 2. Unstatistically significant differences emerged between the two groups at the baseline. Of the patients who underwent TR-PB, 33 (27.3%) were not biopsy-naive: Three (2.5%) were following an active surveillance protocol due to a previous finding of suspicious low-risk PCa, while the residual 30 patients (24.8%) were persistently suspicious for PCa (elevated PSA values or clinical/imaging progression), despite one or more previous negative prostate biopsies (Table 3).

The median age at the time of PB was 64 years, the median PSA value was 7.3 ng/mL, the median PV was 57.8 mL, and the median number of cores was 13 (Table 1). Concerning complications after biopsy, 35 patients (26.9%) experienced early side effects in the first week after the procedure (Table 4). Only seven patients (5.8%) experienced late side effects in the second week after TR-PB (Table 4). There were no significant differences concerning anticoagulant/antiplatelet therapy between the two groups.

Comparison of the complications after TR-PB with a US standard technique and TR-PB with an MRI-US cognitive technique showed significantly more early side effects, especially in terms of rectal bleeding, with a total of 10.2% vs. 1.6%. Concerning late side effects, there was a non-significant difference between the two groups: 4.8% for the MRI-guided plus the standard vs. 6.7% for the standard only. Four patients (3.3%) were evaluated for AUR and required temporary catheterization for a median of 4.5 days (IQR: 2–9) after accessing the emergency room. Among these, only one patient had a readmission to the emergency room for an evaluation of catheter placement four days after the catheterization.

Only one of the patients (0.85%) required hospitalization for rectal bleeding and needed blood transfusions.

UTIs treated with antibiotic therapy occurred in seven patients (5.8%) and were managed at home with oral antibiotic therapy.

## 4. Discussion

To our knowledge, this is the first case series detailing outcomes of the TR-PB approach in kidney transplant recipients. The management of PCa has changed over the last 20 years, starting new recommendations for pre-kidney transplant patients [7]. The guidelines for pre-KT candidates mention that Grade Group 1 PCa is not a contraindication to KT and should not delay the waiting time for transplantation [3]. However, a prostate MRI is recommended to be sure that all zones are correctly sampled. Hevia et al. [8] reported, in a systematic review of 41 studies, outcomes of patients diagnosed with PCa following KT. Of the 319 men, 82%, 17%, and 6% were managed with radical prostatectomy, external beam radiation therapy, or brachytherapy, respectively. They observed that the mean age, PSA, and distribution of the Gleason score was like patients undergoing radical prostatectomy in the general population, which is yet further evidence that immunotherapy does not adversely influence the biology of PCa following renal transplantation. Although long-term data are lacking, oncological control assessed by biochemical recurrence rates appear to parallel those observed in the general population when stratified according to the baseline Gleason score [9].

TR-PB US-guided is the most commonly used technique for PCa diagnosis. With the introduction of the MRI-guided PB, a targeted biopsy of suspicious zones is often performed on biopsy-naìve men. However, there has been a remarkable intensification in infectious complication rates after PBs using the transrectal technique [10]. Thus, there is growing attention to the transperineal technique for PCa identification. The latest multi-institutional study declared that the use of the transperineal MRI-targeted PB improved the detection of csPCa relative to the transrectal MRI-targeted PB, in particular, for tumors located in the anterior, apex and transition zone [11].

There is a discussion regarding a higher rate of complications related to TR-PB and the transperineal prostate biopsy (TP-PB). Numerous authors have mentioned that TRPB and TPPB show similar rates of minor adverse events, but the discrepancy becomes marked when examining infectious complications [12,13]. TR-PB appears to have a higher risk of infection because the needle biopsy is carried through the rectum, and bacteria from the rectal mucosa (usually E. coli) migrate to the prostate, urinary system or blood vessels. Historically, Fluoroquinolones have been the agents of choice for prophylaxis due to their good pharmacokinetics in prostatic tissue [14]. However, the European Urological Association (EAU) banned Fluoroquinolones from antibiotic prophylaxis in urological surgical treatments due to their increase resistance [15].

Infection still remains a problem in organ transplantation. While most infections in the first month after transplantation are generally healthcare–associated infections, infections after 6–12 months are community-acquired infections. For these reasons, we never perform a prostate biopsy before 12 months after the KT [16]. In this context, Ceftriaxone is an attractive alternative due to its broad-spectrum activity against many Gram-positive and Gram-negative bacteria [13]. It is also effective against Escherichia coli, the most common pathogen after TR-PB [14]. All men with TR-PB should receive antimicrobial prophylaxis and be warned about the increasing risk of infection. We usually administer one prophylactic dose of Fosfomycin Trometamol (3 g), two hours before the biopsy. In cases where an allergy to Fosfomycin was reported, we treat the patients with one prophylactic dose of Ciprofloxacin (500 mg), two hours before the procedure.

The EAU Guidelines recommend switching from TR-PB to TP-PB [17]. On the other hand, the American Urological Association suggests both a transrectal and the transperineal approach, due to a lack of evidence on the infectious risk [13].

Some zones are easier to biopsy than others (the base and posterior surface), due to the transrectal techniques. The TR-PB biopsy gives easy access to the prostate base but because of the anatomy of the gland, anterior-apex lesions may be difficult to biopsy because of the depth needed to reach them, as well as because of the urethra, which can be penetrated in the procedure. The biopsy of the apical zone has great importance, since it can increase the overall prostate cancer detection by 7.8% [14]. Some studies recommend sampling the anterior apical region of the gland using a TP-BP instead of TR-PB, both for easier access and for lower UTIs [10,14].

In this setting, our study aims to retrospectively evaluate the impact of a standardized clinical pathway for TR-PB, focusing on post-procedural complications in these fragile patients in order to assess whether this technique could still be a valuable possibility for the detection of PCa or whether we should abandon it. Our findings indicate that TR-PB is generally well-tolerated (only 2.5% of cases required post-procedural analgesics), and related with a low risk of major adverse events. We observed a 28.9% Clavien–Dindo grade ≤ 2 of early side effect rates (mostly rectal bleeding and other minor hematuria), with a very low rate of hospital re-admission in the emergency room for AUR (3.3%); only one man required hospitalization for rectal bleeding, and there were no major complications (such as sepsis). Of note, our data are at least comparable to those available in the literature. Our results are consistent with Guo et al. [18], which observed no difference between TP-PB and TR-PB, considering the minor complication rate (44.9% vs. 41.0%). In this report, major adverse events comprising high fever (0% vs. 1.2%), sepsis (0% vs. 0.6%) and severe rectal bleeding (0% vs. 1.2%) happened at a significantly higher rate in the TR-PB group (4.3%) compared to TP-PB (0.6%). Similarly, in the meta-analysis by Xue et al. [4] comparing TR-PB vs. TP-PB, no significant differences in terms of hematuria (20.6% vs. 17.1%), UTIs (0.4% vs. 0.4%), AUR (3.8% vs. 2.4%) and hematospermia (0.7% vs. 1.2%) were reported. The presence of blood in the ejaculation fluid is the most frequently reported side effect after prostate biopsies, in some studies reaching an incidence of up to 93% in the general population of patients [10]. In the literature, the problem was also described up to five weeks after the procedure [12]. As regards to this last common adverse event (hemospermia), it was not taken into consideration as an adverse event in our study, because at the time of discharge, the patients were advised to abstain from sexual activity for at least two weeks. As regards to hospitalization rates, data in the literature report that they vary depending on the study and the approach used for the biopsy. Anastasiadis et al. [19] reported that the hospitalization rate was 3.7% due to biopsy-related adverse events. Independent predictors of complications requiring hospitalization were age and comorbidities. In a retrospective analysis, Nam and colleagues found that the overall hospitalization rate was 1.4%, with no significant differences by age [20]. In our study, only one patient was hospitalized after TR-PB because they needed blood transfusions for rectorrhagia. Ghani et al. [21] and Berger et al. [22] found a significantly higher rate of rectorrhagia linked with the number of cores (1.3% and 2.3%, respectively). In this retrospective study, the occurrence of rectal bleeding was 10.2% in the standard TR biopsy group and 1.6% in the cognitive MRI-guided biopsy group in the first week, of which zero had late side effects. These data in our study could correlate not only to the higher number of cores, but also to the higher percentage of patients affected by hemorrhoids in G1 (32.2%) compared to G2 (14.5%). In the literature, rectal bleeding is the most frequently reported complication after TR-PB, and resolves spontaneously. Due to the transrectal approach of the biopsy, pathologies such as hemorrhoids and anal fissures can generate problems [21,22,23]. However, the TR-PB is not an emergency technique, so patients should first treat the hemorrhoids; some may respond to pharmaceutical treatment. Either way, a biopsy should be postponed for a period. mpMRI might be a good alternative for immediate results; however, a biopsy will still be needed for histologic confirmation.

Another complication, AUR, occurs in 0.4% to 6% of patients, depending on the study. In the literature, the occurrence of AUR has been observed to be slightly higher after the TP-PB approach (1.7–11.1%). Pepe et al. [23] described that the incidence of AUR increased with the number of cores taken. In our study, we had six (4.9%) cases of AUR and all were distributed equally between two groups (4 in the first week and 2 in the second week after the procedure). In a general population, an exacerbation of the lower urinary tract symptoms may also occur after the biopsy, particularly in men with an enlarged prostate, but AUR is infrequent. However, our retrospective study had several limitations; first, there was a small number of patients in the cohort, as well as missing data from six patients (4.9%) for the follow-up, which were thus excluded. Second, a bias exists due to a flaw in the sample selection process; the technique approach was selected by the urologist based on patient characteristics and according to his experience. Third, a multivariate analysis of risk factors for early and late postoperative complications was not performed in this study.

## 5. Conclusions

Prostate cancer stands out as one of the most prevalent tumors affecting men, and PB is considered the gold standard for diagnosing this condition. While a shift towards the TP-PB approach is recommended and desirable, it is noteworthy that TR-PB continues to be a secure and cost-effective method with a low risk of severe complications. Nevertheless, individuals with a transplanted organ often present a complex medical history, numerous comorbidities, polypharmacy, and specific needs related to transplantation. Providing care for organ transplant recipients during invasive procedures such as PB demands specialized knowledge and collaboration with a variety of healthcare professionals, potentially reducing complication rates and enhancing the detection of clinically significant prostate cancer. Acknowledging the limitations of this study, we can affirm that TR-PB can be a safe procedure with a low risk of severe adverse events when performed by skilled specialists and with a standardized procedural pathway, even in a complex patient setting such as with a transplant recipient. However, overall, men undergoing biopsy are generally healthier than the general population, and biopsy-related mortality is extremely rare. Further prospective randomized trials are necessary to compare the safety of this approach in the transplant patient population. This is essential in order to prepare more detailed answers to the ongoing debate on the strengths and limitations of this procedure extended to sets of “fragile patients”.

## Figures and Tables

**Table 1:** Clinical characteristics of patients undergoing transrectal prostate biopsy procedures.

**Patients’ Characteristics**	**Group 1** **TR-PB US** **Standard** ***n*: 59**	**Group 2** **TR-PB MRI-US** **Cognitive** ***n*: 62**	***p*** **Value**
Age (years):	63.5 (47.7–78.9)	65.2 (51.3–77.7)	NS
1st degree positive family history, *n* (%):	9 (15.2)	11 (17.7)	NS
Race, *n* (%):			
African	1 (1.7)	0	NS
American	0	1 (1.6)	NS
Asian	1 (1.7)	1 (1.6)	NS
Caucasian	57 (96.6)	60 (96.8)	NS
PSA levels ng/mL, median (IQR):	6.7 (2.8–15.3)	7.9 (3.2–17.4)	NS
Prostate volume mL, median (IQR):	58.3 (22–84)	57.2 (21–87)	NS
Positive/suspicious DRE, *n* (%):	34 (57.6)	38 (61.3)	NS
Comorbidities, *n* (%):			
Diabetes mellitus	8 (13.5)	15 (24.2)	<0.001
Cardiovascular disease (not hypertension)	11 (18.6)	13 (20.9)	NS
Hemorrhoids	19 (32.2)	9 (14.5)	<0.001
Antiplatelet/anticoagulant drugs, *n* (%):			
Suspended before TR-PB	19 (32.2)	21 (33.9)	NS
Not suspended before TR-PB	11 (18.6)	13 (20.9)	NS

NS: not significant; TR-PB: Transrectal Prostate Biopsy; US: Ultrasound; MRI: Magnetic Resonance Imaging; DRE: Digital Rectal Examination.

**Table 2:** Patients and transplant features.

**Transplant and Kidney Failure Features**	**Group 1** **TR-PB US** **Standard** ***n*: 59**	**Group 2** **TR-PB MRI-US** **Cognitive** ***n*: 62**	***p*** **Value**
Renal function at TR-PB, median (IQR):			
Creatinine mg/dL	1.7 (1.19–2.41)	1.6 (1.24–2.39)	NS
eGFR mL/min	46.8 (31.6–64.5)	45.8 (32.3–63.7)	NS
Cause of renal failure, *n* (%):			
Chronic glomerulonephritis	23 (39)	21 (33.9)	<0.001
APKD	14 (23.7)	16 (25.8)	NS
Diabetic nephropathy	8 (13.5)	15 (24.2)	<0.001
Nephrosclerosis	4 (6.8)	6 (9.7)	NS
Chronic pyelonephritis	1 (1.7)	2 (3.2)	NS
Congenital renal dysplasia	0	1 (1.6)	NS
Others	1 (1.7)	1 (1.6)	NS
Number of kidney transplants, *n* (%):			
1	57 (96.6)	59 (95.1)	NS
2	2 (3.4)	3 (4.9)	NS
Type of first transplant, *n* (%):			
Single cadaver	57 (96.6)	59 (95.2)	NS
Single living donor	1 (1.7)	2 (3.2)	NS
Double cadaver	1 (1.7)	1 (1.6)	NS

NS: not significant; TR-PB: Transrectal Prostate Biopsy; US: Ultrasound; MRI: Magnetic Resonance Imaging; eGFR: Estimated Glomerular Filtration Rate; APKD: Autosomal Dominant Polycystic Kidney Disease.

**Table 3:** Features of the biopsy and cancer detection rate of two transrectal approaches.

**Features of the Biopsy and Cancer Detection Rate**	**Group 1** **TR-PB US** **Standard** ***n*: 59**	**Group 2** **TR-PB MRI-US** **Cognitive** ***n*: 62**	***p*** **Value**
Indication for biopsy, *n* (%):			
Biopsy naìve	43 (72.9)	45 (72.6)	NS
Surveillance	2 (3.4)	1 (1.6)	NS
Prior negative biopsy	14 (23.7)	16 (25.8)	NS
Location of index lesion (MRI/US), *n* (%):			
Peripheral zone	53 (89.9)	51 (82.3)	NS
Transition zone	6 (10.1)	11 (17.7)	<0.001
PI-RADS, *n* (%):			
<3	-	0	
3	-	37 (59.7)	
4–5	-	25 (40.3)	
Total number of targeted cores, *n*:	236	279	NS
Number of positive targeted cores, *n* (%):	65 (27.5)	97 (34.7)	<0.001
Biopsy ISUP grade, *n* (%):			
GG1	14 (23.7)	16 (25.8)	NS
GG2	6 (10.1)	8 (12.9)	NS
GG3	3 (5.1)	1 (1.6)	NS
GG4	1 (1.7)	1 (1.6)	NS
GG5	0	0	NS
ASAP, *n* (%):	2 (3.4)	1 (1.6)	NS
HG-PIN, *n* (%):	1 (1.7)	2 (3.2)	NS

NS: not significant; TR-PB: Transrectal Prostate Biopsy; US: Ultrasound; MRI: Magnetic Resonance Imaging; ASAP: Atypical Small Acinar Proliferation; HG-PIN: High-grade Prostatic Intraepithelial Neoplasia.

**Table 4:** Side effects according to the type of approach and timing of onset.

**Side Effects**	**Group 1** **TR-PB US** **Standard** ***n*: 59**	**Group 2** **TR-PB MRI-US** **Cognitive** ***n*: 62**	***p*** **Value**
Early side effects (days 1–7), *n* (%):			
Pain requiring analgesics	2 (3.4)	1 (1.6)	NS
Hematuria	7 (11.9)	9 (14.5)	NS
Rectal bleeding	6 (10.2)	1 (1.6)	<0.001
Urinary tract infections	3 (5.1)	2 (3.2)	NS
Acute urinary retention	2 (3.4)	2 (3.2)	NS
Sepsis	0	0	NS
Late side effects (days 7–15), *n* (%):			
Pain requiring analgesics	0	0	NS
Hematuria	2 (3.4)	1 (1.6)	NS
Rectal bleeding	0	0	NS
Urinary tract infections	1 (1.7)	1(1.6)	NS
Acute urinary retention	1 (1.7)	1 (1.6)	NS
Sepsis	0	0	NS

NS: not significant; TR-PB: Transrectal Prostate Biopsy; US: Ultrasound; MRI: Magnetic Resonance Imaging.

## Data Availability

The original contributions presented in the study are included in the article. Further inquiries can be directed to the corresponding author.

## References

[1] Becher E., Wang A., Lepor H. (2019). Prostate Cancer Screening and Management in Solid Organ Transplant Candidates and Recipients. Rev. Urol..

[2] Siegel R.L., Miller K.D., Jemal A. (2020). Cancer statistics, 2020. CA Cancer J. Clin..

[3] KDIGO Kdigo Clinical Practice Guideline on the Evaluation and Management of Candidates for Kidney Transplantation: Public Review Draft. October 2018. https://kdigo.org/wp-content/uploads/2018/08/KDIGO-Txp-Candidate-GL-Public-Review-Draft-Oct-22.pdf.

[4] Xue J., Qin Z., Cai H., Zhang C., Li X., Xu W., Wang J., Xu Z., Yu B., Xu T. (2017). Comparison between transrectal and transperineal prostate biopsy for detection of prostate cancer: A meta-analysis and trial sequential analysis. Oncotarget.

[5] Rapisarda S., Bada M., Crocetto F., Barone B., Arcaniolo D., Polara A., Imbimbo C., Grosso G. (2020). The role of multiparametric resonance and biopsy in prostate cancer detection: Comparison with definitive histological report after laparoscopic/robotic radical prostatectomy. Abdom. Radiol..

[6] Dell’Atti L. (2015). The best prostate biopsy scheme is dictated by the gland volume: A monocentric study. Eur. Rev. Med. Pharmacol. Sci..

[7] Haeuser L., Nguyen D.D., Trinh Q.D. (2020). Prostate cancer and kidney transplantation—Exclusion or co-existence?. BJU Int..

[8] Hevia V., Boissier R., Rodríguez-Faba Ó., Fraser-Taylor C., Hassan-Zakri R., Lledo E., Regele H., Buddde K., Figueiredo A., Olsburgh J. (2018). Management of Localised Prostate Cancer in Kidney Transplant Patients: A Systematic Review from the EAU Guidelines on Renal Transplantation Panel. Eur. Urol. Focus.

[9] Bratt O., Drevin L., Prütz K.-G., Carlsson S., Wennberg L., Stattin P. (2020). Prostate cancer in kidney transplant recipients—A nationwide register study. BJU Int..

[10] Mate K., Nedjim S., Bellucci S., Boucault C., Ghaffar N., Constantini T., Marvanykovi F., Vestris P.G., Sadreux Y., Laguerre M. (2023). Prostate biopsy approach and complication rates. Oncol. Lett..

[11] Gentile F., La Civita E., Della Ventura B., Ferro M., Cennamo M., Bruzzese D., Crocetto F., Velotta R., Terracciano D. (2022). A Combinatorial Neural Network Analysis Reveals a Synergistic Behaviour of Multiparametric Magnetic Resonance and Prostate Health Index in the Identification of Clinically Significant Prostate Cancer. Clin. Genitourin. Cancer.

[12] Shen P.-F., Zhu Y.-C., Wei W.-R., Li Y.-Z., Yang J., Li Y.T., Li D.M., Wang J., Zeng H. (2012). The results of transperineal versus transrectal prostate biopsy: A systematic review and meta-analysis. Asian J. Androl..

[13] Alberti A., Nicoletti R., Polverino P., Rivetti A., Dibilio E., Resta G.R., Makrides P., Caneschi C., Cifarelli A., D’Amico A. (2023). Morbidity of Transrectal MRI-Fusion Targeted Prostate Biopsy at a Tertiary Referral Academic Centre: An Audit to Guide the Transition to the Transperineal Approach. Cancers.

[14] Roberts M.J., Scott S., Harris P.N., Naber K., Wagenlehner F.M.E., Doi S.A.R. (2018). Comparison of fosfomycin against fluoroquinolones for transrectal prostate biopsy prophylaxis: An individual patient-data meta-analysis. World J. Urol..

[15] Bonkat G., Pilatz A., Wagenlehner F. (2019). Time to Adapt Our Practice? The European Commission Has Restricted the Use of Fluoroquinolones since March 2019. Eur. Urol..

[16] Wang J.H., Skeans M.A., Israni A.K. (2016). Current Status of Kidney Transplant Outcomes: Dying to Survive. Adv. Chronic Kidney Dis..

[17] Mottet N., Cornford P., van den Bergh R.C.N., Briers E., Eberli D., De Meerleer G., De Santis M., Gillessen S., Grummet J., Expert Patient Advocate (European Prostate Cancer Coalition/Europa EOMO) (2023). EAU Guidelines on Prostate Cancer.

[18] Guo L.-H., Wu R., Xu H.-X., Xu J.-M., Wu J., Wang S., Bo X.W., Liu B.J. (2015). Comparison between Ultrasound Guided Transperineal and Transrectal Prostate Biopsy: A Prospective, Randomized and Controlled Trial. Sci. Rep..

[19] Anastasiadis E., van der Meulen J., Emberton M. (2015). Hospital admissions after transrectal ultrasound-guided biopsy of the prostate in men diagnosed with prostate cancer: A database analysis in England. Int. J. Urol..

[20] Nam R.K., Saskin R., Lee Y., Liu Y., Law C., Klotz L.H., Loblaw D.A., Trachtenberg J., Stanimirovic A., Simor A.E. (2013). Increasing hospital admission rates for urological complications after transrectal ultrasound guided prostate biopsy. J. Urol..

[21] Ghani K.R., Dundas D., Patel U. (2004). Bleeding after transrectal ultrasonography-guided prostate biopsy: A study of 7-day morbidity after a six-, eight- and 12-core biopsy protocol. BJU Int..

[22] Berger A.P., Gozzi C., Steiner H., Frauscher F., Varkarakis J., Rogatsch H., Bartsch G., Horninger W. (2004). Complication rate of transrectal ultrasound guided prostate biopsy: A comparison among 3 protocols with 6, 10 and 15 cores. J. Urol..

[23] Pepe P., Aragona F. (2013). Morbidity after transperineal prostate biopsy in 3000 patients undergoing 12 vs 18 vs more than 24 needle cores. Urology.

